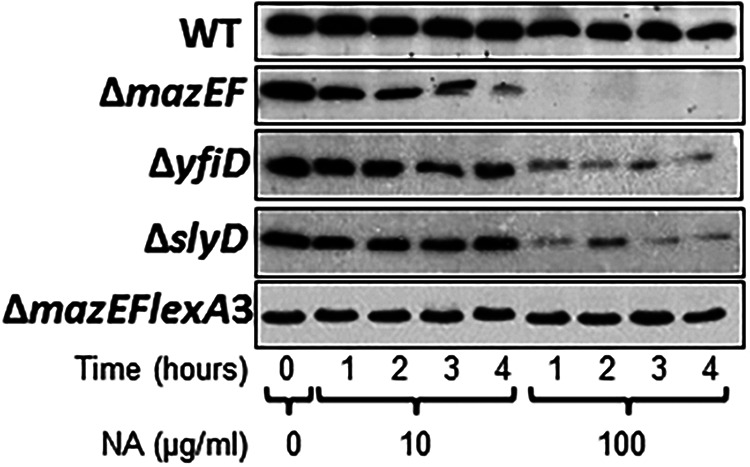# Correction for Erental et al., “Apoptosis-Like Death, an Extreme SOS Response in Escherichia coli”

**DOI:** 10.1128/mBio.03040-20

**Published:** 2020-12-15

**Authors:** Ariel Erental, Ziva Kalderon, Ann Saada, Yoav Smith, Hanna Engelberg-Kulka

**Affiliations:** a Department of Microbiology and Molecular Genetics, Institute for Medical Research Israel-Canada (IMRIC), The Hebrew University-Hadassah Medical School, Jerusalem, Israel; b Monique and Jacques Robo Department of Genetic Research, Hadassah-Hebrew University Medical Center, Jerusalem, Israel; c Department of Genetic and Metabolic Diseases, Hadassah-Hebrew University Medical Center, Jerusalem, Israel; d Genomic Data Analysis Unit, The Hebrew University-Hadassah Medical School, The Hebrew University of Jerusalem, Jerusalem, Israel

## AUTHOR CORRECTION

Volume 5, no. 4, e01426-14, 2018, https://doi.org/10.1128/mBio.01426-14. In the Western blot analysis experiment that appeared in Fig. 4 of the original paper, an incorrect panel for the Δ*mazEFlexA3* strain was pasted into the figure during our editing process. We provide here a corrected Fig. 4 including the correct Δ*mazEFlexA3* panel.

**Figure:**